# A retrospective study of anthrax on the Ghaap Plateau, Northern Cape province of South Africa, with special reference to the 2007–2008 outbreaks

**DOI:** 10.4102/ojvr.v84i1.1414

**Published:** 2017-09-28

**Authors:** Ayesha Hassim, Edgar H. Dekker, Charles Byaruhanga, Tommy Reardon, Henriette van Heerden

**Affiliations:** 1 Department of Veterinary Tropical Diseases, University of Pretoria, South Africa; 2 Department of Agriculture, Forestry and Fisheries, South Africa

## Abstract

Anthrax is a zoonotic disease caused by the gram-positive, endospore-forming and soil-borne bacterium *Bacillus anthracis*. When in spore form, the organism can survive in dormancy in the environment for decades. It is a controlled disease of livestock and wild ungulates in South Africa. In South Africa, the two enzootic regions are the Kruger National Park and the Ghaap Plateau in the Northern Cape province. Farms on the Plateau span thousands of hectares comprising of wildlife – livestock mixed use farming. In 2007–2008, anthrax outbreaks in the province led to government officials intervening to aid farmers with control measures aimed at preventing further losses. Because of the ability of the organism to persist in the environment for prolonged periods, an environmental risk or isolation survey was carried out in 2012 to determine the efficacy of control measures employed during the 2007–2008, anthrax outbreaks. No *B. anthracis* could be isolated from the old carcass sites, even when bone fragments from the carcasses were still clearly evident. This is an indication that the control measures and protocols were apparently successful in stemming the continuity of spore deposits at previously positive carcass sites.

## Introduction

The Northern Cape province (NCP) is in South Africa, situated 30° S, 22° E. It is the largest province in South Africa spanning 372 889 km^2^. A dolomitic escarpment elevates the mid-eastern border of the province, extending 275 km to the south west, known as the Ghaap Plateau (Partridge et al. 2010; Smit 1978). The province is divided into two ecological areas, namely the Savannah Biome in the north-eastern half of the province while the south-western half hosts the rare and more arid Nama Karoo Biome (Fourie & Roberts 1973). The province contains a number of state, provincial and privately owned wildlife conservancy areas. The NCP also services the Kgalagadi Transfrontier Park bordering Botswana and the Richtersveld Transfrontier Park bordering Namibia (http://www.sanparks.co.za/conservation). Because of this predominating savannah in the eastern part of the province, the majority of the remaining land is utilised for extensive farming of sheep, cattle and mixed farming which includes wild game (http://www.southafrica.info/about/geography/northern-cape.htm).

The NCP has alkali, phosphorus deficient soils which lead to pica in grazers and browsers in the form of osteophagia and geophagia (Boyazoglu 1973; De Vos & Turnbull 2004; Theiler 1912). This animal behavioural characteristic has resulted in a variety of infections with similar pathologies of which ‘lamsiekte’ (botulism, *Clostridium botulinum*, ‘miltsiekte’ (anthrax, *Bacillus anthracis*) and ‘stijfsiekte’ (Three Day Sickness, *rhabdovirus*) are amongst the most common (Theiler 1912; Viljoen, Curson & Fourie 1928). Both anthrax and botulism are caused by soil-borne, spore-forming and toxin-producing bacteria. Carcasses are typically observed to have an opisthotonic form with oedema or protruding of the tongue for both diseases (Edmonds 1922; Theiler 1912; Van der Lugt et al. 1995). Anthrax and botulism have been observed to occur on the same farm, even simultaneously at times and this has been a cause for misdiagnosis in the past (Kriek & Odendaal 1994; Theiler 1912, 1927; Viljoen et al. 1928).

Anthrax is an acute or peracute zoonotic disease predominantly affecting livestock and wild ungulates with episodic spill over to humans and carnivores. It is characterised by oedema, sudden death syndrome, black eschars and haemorrhaging from the orifices (Turnbull 2008). The disease is caused by the gram-positive, aerobic and endospore-forming bacterium *B. anthracis*. Vegetative *B. anthracis* cells have a distinct encapsulated, *s*quare ended ‘box-shaped’ appearance on Giemsa stained blood smears (Hugh-Jones & De Vos 2002; Turnbull 2008), which is a means of distinguishing them from other gram-positive rod shaped bacteria microscopically (Theiler 1912). The use of selective media and morphological selection are typically employed for bacteriologic isolation of *B. anthracis* followed by testing for sensitivity to Gamma phage and penicillin as well as verification of virulence factors as added confirmation for the bacterium and differentiation from closely related *Bacillus cereus sensu lato* group organisms (Knisely 1966; Turnbull 1999).

Infection of a host can be through ingestion, inhalation or cutaneous. Once an infected animal has died, its carcass becomes a potential site of infection for the next host (Dragon et al. 2005). Anthrax is an World Organisation for Animal Health (OIE) reportable disease and opening of carcasses is strictly prohibited (Turnbull 2008). Sporulation is triggered by nutrient shortages and exposure to oxygen (Sterne 1937; Turnbull 2008). Bacterial spore counts are higher where bloody discharge from the orifices and bodily fluids from the carcass soak the ground (Bellan et al. 2013). Sporulation also takes place when a carcass is opened by scavengers such as vultures (*Gyps* spp./*Trigonoceps occipitalis*/*Torgos tracheliotos*), crows (*Corvus* spp.), jackal (*Canis* spp.) or hyena (*Crocuta crocuta*) (Hugh-Jones & De Vos 2002; Turnbull 2008). Blowflies are considered as mechanical vectors of anthrax in the Kruger National Park (KNP) because they feed on a carcass and then deposit *B. anthracis* laden regurgitate on vegetation around the carcass. This contaminated vegetation may then be a potential source of infection to susceptible kudus (*Tragelaphus strepsiceros*) and other browsers or grazers (Blackburn et al. 2014; Braack & De Vos 1990; De Vos & Turnbull 2004; Hugh-Jones & De Vos 2002). Spores in the environment have been recovered by De Vos (1990) from bones during archaeological excavations at a site in KNP that were estimated to be 200 ± 50 years old.

To reduce such spore inoculum in the environment incineration of anthrax carcasses and burial have been the preferred method and in accordance with WHO and OIE guidelines internationally (Turnbull 2008). Treating anthrax carcasses with 10% formalin would kill the bacteria, deter scavengers that would open the carcass and decrease spread by flies, but remains controversial because of the health and safety issues related to its handling. Turnbull (2008) also proposed covering or wrapping carcasses in plastic or tarpaulins to keep the skin intact and reduce vegetative *Bacilli* through putrefaction and thus anthrax spores in the environment. Other control methods include covering the carcass in thorns to deter animals from eating at the site, as well as the use of insecticides to inhibit the role of flies and carrion insects on the carcass (Williams & Barker 2008; Williams & Richardson 1984).

The two anthrax-enzootic areas in South Africa (Figure 1) are the NCP and KNP (Bengis et al. 2002; De Vos & Turnbull 2004; Hugh-Jones & De Vos 2002; Smith et al. 2000). While mandatory vaccination schemes have virtually abolished anthrax in livestock in most of the country, these areas remain enzootic because of the predominance of wildlife conservancies and game farms (Gilfoyle 2006; Turnbull 2008). A large number of animals usually succumb to the disease before it is noticed because of the short incubation period and the large areas involved. Kudus are typical fatalities of anthrax, but in 2008 large numbers of antelope and equids were also affected in the NCP outbreaks. The outbreak gained momentum from 2007, and by the end of March 2008, had resulted in the deaths of thousands of heads of game and economic losses to the farmers of the Ghaap Plateau amounting to millions of South African rand (Visagie 2008). It was the largest recorded outbreak in the region in recent history and the Department of Agriculture Fisheries and Forestry in South Africa mobilised national and provincial state veterinary services to aid in diagnosis, surveillance and control measures in order to stem the outbreaks (Nduli 2009; Visagie 2008). The aims of this study were (1) to report the 2007/2008 *B. anthracis* outbreaks in the NCP, (2) to determine the *B. anthracis* spore concentrations from bone and environmental samples during the 2007–2008 NCP anthrax outbreaks using bacteriological methods and (3) to compare the spore concentrations from bone and soil collected at the same sites in 2012 to determine the spore endurance and/or efficacy of the control measures employed during the 2007/2008 outbreaks in reducing the inoculum in the environment.

**FIGURE 1:**
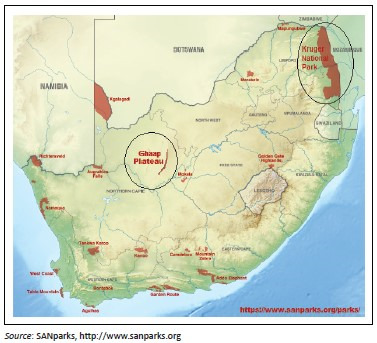
A modified map of South Africa detailing the National Parks. The two circled areas represent the anthrax-enzootic areas (Kruger National Park and the Ghaap Plateau).

## Materials and methods

### Observations, sample collection and control measures during 2007–2008 anthrax outbreaks

The first cases of the 2007–2008 anthrax outbreaks in NCP were reported in November 2007 at Doringbult farm (Figure 2) after heavy spring rainfall during mid-August – November 2007. Later that month, the skeletal remains of one female kudu (NC/17) on Grootsalmonsfontein and two female kudus (NC/27 and NC/28) on the adjacent farm Dikbosch were recovered, all of which were gestating (EH Dekker pers. comm., February 2013; Nduli 2009). The first isolated cases of anthrax from kudu were identified in the latter farm Dikbosch (Figure 2). In February 2008, five more kudu carcasses (both males and females) were discovered on Dikbosch and Kleinsalmonsfontein farms and by the following month anthrax cases included zebra, wildebeest, impala, sheep and kudu along the length of the Ghaap escarpment (Figure 2).

**FIGURE 2:**
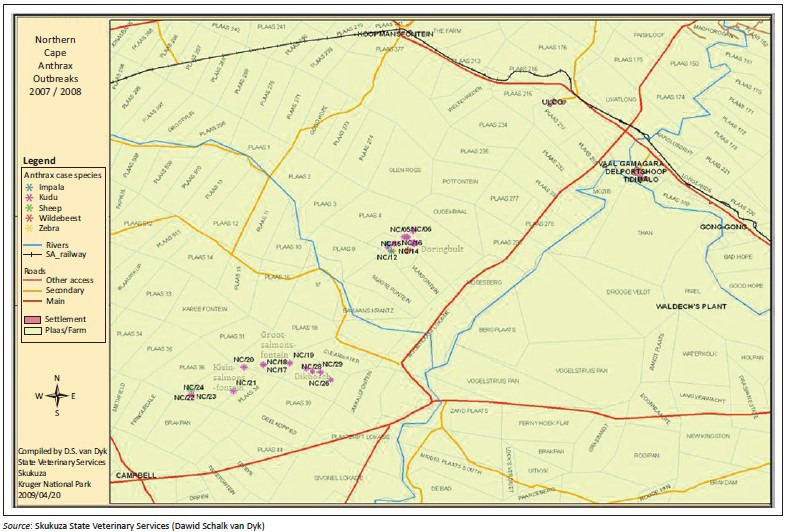
Anthrax-positive carcass sites indicating the distribution of susceptible species discovered on farms during the 2007–2008 anthrax outbreaks in the Ghaap Plateau in the Northern Cape province, South Africa.

After a kudu at Doringbult was confirmed suspicious for anthrax from a Giemsa stained blood smear, more comprehensive samples were collected for diagnostics. KNP (Skukuza Veterinary Services) and Northern Cape province Veterinary Services visited farms to aid farmers in the diagnosis and control of the outbreak. The condition of the carcasses was documented along with collection of blood smear and bone samples (mandibular, orbital, rib, vertebra, femur and/or pelvic bones if possible). Soil samples were collected from under the head, abdomen and tail of the carcass for diagnostic purposes. Environmental samples such as crow faeces and bone fragments were collected from carcass sites where evidence of scavenger activity was visible (Table 1). Louse flies (*Hippobosca rufipes*) were observed and collected from recently dead kudu carcasses at Clearwater and Klipfontein (Table 1, Figure 3a). Random soil samples were also collected from farms at the top of the plateau and pans at the base of the escarpment to determine spore counts in areas not contaminated by fresh carcasses (Table 1).

**FIGURE 3:**
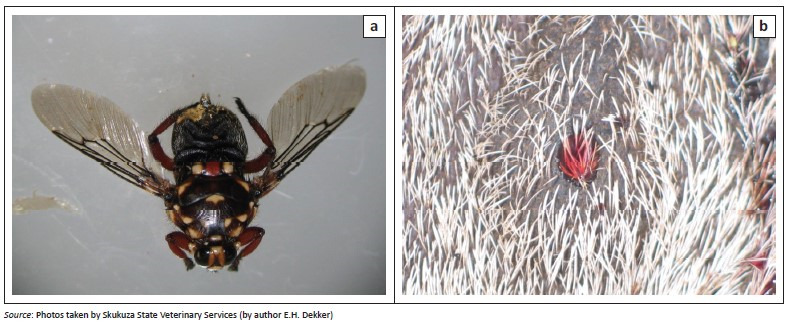
(a) Louse fly (*Hippobosca rufipes*) collected from an anthrax-positive carcass from Clearwater, South Africa, during the 2008 outbreaks. (b) Site on anthrax-positive carcass where *Hippobosca rufipes* had fed.

**TABLE 1:** Anthrax-positive carcasses (based on Giemsa stained blood smears) and environmental samples collected from farms along the Ghaap Plateau in the Northern Cape province, South Africa, during outbreaks in 2007–2008 indicating control measures implemented by farm owners and State Veterinary Services.

Sample (Processing N°)	Location	Species	Sex	Age	Date	Actions and observations	Latitude	Longitude
NC/01	Doringbult	Kudu	Male	18 Months	Apr. 2008	Covered with tarp.	NR	NR
NCP/33	Random Soil Sample taken at the highest and lowest point of the Doringbult farm ‘drainage basin’	-	-	-	-	-	−28.491415	24.05425667
NC/02	Doringbult	Impala	Male	18 Months	Apr. 2008	Covered with tarp.	−28.50074333	24.05014667
NC/03	Doringbult	Kudu	Male	12 Months	Apr. 2008	Covered with tarp but only put over a few days after animal’s death.	−28.49014167	24.04442
NC/04	Doringbult	Kudu	Female	Adult	Apr. 2008	Covered with tarp. Head was open. Visible hole in abdomen.	−28.48999	24.04443833
NC/05	Doringbult	Kudu	Female	Adult	Apr. 2008	Covered with tarp. Head was open.	NR	NR
NC/06	Doringbult	Kudu	Female	Adult	Apr. 2008	Covered with tarp. Unopened. Only soil was collected.	−28.49002333	24.04442333
NC/07	Doringbult	Kudu	Male	Adult	Apr. 2008	Covered with tarp. Consumed by blowflies. Blowflies collected.	−28.48682833	24.03966833
NC/08	Doringbult	Kudu	Female	12 Months	Apr. 2008	Covered with tarp. Carcass still wet. High number of maggots.	−28.48682833	24.03966833
NC/09	Doringbult	Kudu	Female	Adult	Apr. 2008	Covered with tarp. Carcass still wet. Skin partially rotted. High maggot activity.	−28.3038	24.02727
NC/10	Doringbult	Kudu	Male	Adult	Apr. 2008	Covered with tarp. Sprayed with 10% chlorine. Carcass still wet.	−28.48682833	24.03966833
NC/11	Vlakfontein	Impala	Male	18 Months	Apr. 2008	Covered with tarp. Well covered 10% chlorine. No maggots observed.	−28.30417	24.00891
NC/12	Vlakfontein	Zebra	Male	Adult	Apr. 2008	Covered with tarp. Well covered in 10% chlorine. No maggots observed.	−28.30892	24.0118
NC/13	Vlakfontein	Kudu	Female	6 Months	Apr. 2008	Covered with tarp. 10% Chlorine sprayed over and under carcass.	−28.30401	24.01324
NC/14	Vlakfontein	Zebra	Male	Adult	Apr. 2008	Covered with tarp. 10% Chlorine sprayed over and under carcass.	−28.30914	24.0135
NC/15	Vlakfontein	Impala	Male	24 Months	Apr. 2008	Covered with tarp. 10% Chlorine sprayed over and under carcass.	−28.30951	24.0115
NC/16	Vlakfontein	Wildebeest	Male	Adult	Apr. 2008	Covered with tarp. 10% Chlorine sprayed over and under carcass.	−28.30892	24.0118
NCP/23	Grootsalmonsfontein	Kudu	Female	Adult	Apr. 2008	Eyes pecked out. Orbital bone swabbed and remains burned.	−28.76738833	23.77385333
NCP/24	Grootsalmonsfontein	Kudu	Male	Adult	Apr. 2008	Eyes pecked out. Orbital bone swabbed and remains burned.	−28.76600667	23.77385333
NCP/25	Grootsalmonsfontein	Kudu	Male	Adult	Apr. 2008	Eyes pecked out. Orbital bone swabbed.	−28.76672167	23.77427333
NC/17	Grootsalmonsfontein	Kudu	Female	24 Months	Nov. 2007	Complete skeleton treated with 10% formalin. No scavenging apparent. Blowflies collected.	NR	NR
NC/19	Grootsalmonsfontein	Kudu	Male	30 Months	Apr. 2008	Eyes pecked out. Orbital bone swabbed and remains burned.	NR	NR
NC/20	Grootsalmonsfontein	Kudu	Male	Adult	Apr. 2008	No crows or evidence of any. Soil collected.	NR	NR
NC/18	Grootsalmonsfontein	Kudu	Male	36 Months	Apr. 2008	Complete skeleton treated with 10% formalin.	NR	NR
NC/21	Kleinsalmonsfontein	Kudu	Female	Adult	Feb. 2008	Complete skeleton treated with 10% formalin.	−28.70745167	23.85113
NC/22	Kleinsalmonsfontein	Sheep	Female	Adult	Feb. 2008	Treated 10% formalin.	−28.68862167	23.86334833
NC/23	Kleinsalmonsfontein	Kudu	Male	36 Months	Feb. 2008	Treated 10% formalin.	−28.67122667	23.88506167
NC/24	Kleinsalmonsfontein	Kudu	Female	Adult	Feb. 2008	Complete skeleton treated with 10% formalin.	−28.65584	23.90015833
NC/25	Dikbosch	Kudu	Male	12 Months	Feb. 2008	Complete skeleton treated with 10% formalin.	-	-
NC/27	Dikbosch	Kudu	Female	Adult	Nov. 2007	Complete skeleton. Treated with 10% formalin. Bone sampled.	−28.66751167	23.92223833
NC/28	Dikbosch	Kudu	Female	Adult	Nov. 2007	Complete skeleton. Treated with 10% formalin. Bone sampled.	−28.66392167	23.92101167
NC/29	Dikbosch	Kudu	Male	12 Months	Feb. 2008	Complete skeleton. Treated with 10% formalin. Bone sampled.	NR	NR
NCP/27	Dikbosch	Impala	Male	Adult	Apr. 2008	Carcass unopened. Crow faeces and soil collected.	NR	NR
NCP/28	Dikbosch	Kudu	Female	Adult	Apr. 2008	Carcass unopened. *Hippobosca rufipes* flies collected.	NR	NR
NCP/29	Dikbosch	Kudu	Male	Adult	Apr. 2008	Carcass unopened. *Hippobosca rufipes* flies collected.	NR	NR
NCP/30	Dikbosch	Kudu	Male	Adult	Apr. 2008	Carcass unopened. *Hippobosca rufipes* flies collected.	NR	NR
NCP/31	Dikbosch	Kudu	Male	Adult	Apr. 2008	Treated 10% formalin.	NR	NR
NCP/32	Dikbosch	Kudu	Female	Adult	Apr. 2008	Carcass unopened. Crow faeces collected.	NR	NR
NCP/26	Dikbosch	Impala	Male	Adult	Apr. 2008	Carcass unopened. Crow faeces and soil collected.	NR	NR
NCP/01	Clearwater/Schmidtsdrif	Kudu	Male	Adult	22 Apr. 2008	*Hippobosca rufipes* flies.	−28.651815	23.983218
NCP/02	Clearwater/Schmidtsdrif	Kudu	Male	Adult	22 Apr. 2008	*Hippobosca rufipes* flies.	−28.651815	23.983218
NCP/06	Clearwater/Schmidtsdrif	Kudu	Female	Adult	22 Apr. 2008	*Hippobosca rufipes* flies.	−28.65189	24.7479
NCP/14	Clearwater/Schmidtsdrif	Kudu	Male	Adult	22 Apr. 2008	*Hippobosca rufipes* flies.	−28.65159	24.74632
NCP/15	Clearwater/Schmidtsdrif	Kudu	Male	Adult	22 Apr. 2008	*Hippobosca rufipes* flies.	−28.651791	24.751968
NCP/16	Clearwater/Schmidtsdrif	Kudu	Female	Adult	22 Apr. 2008	*Hippobosca rufipes* flies.	−28.65183	24.7501
NCP/17	Clearwater/Schmidtsdrif	Kudu	Male	Adult	22 Apr. 2008	*Hippobosca rufipes* flies.	−28.65183	24.7501
NCP/03	Koppie A (Klipfontein)	Kudu	Male	Adult	22 Apr. 2008	4 X *Hippobosca rufipes* flies.	−28.408715	24.13508833
NCP/04	Koppie B (Klipfontein)	Kudu	Male	Adult	22 Apr. 2008	2 X *Hippobosca rufipes* flies.	−28.40867333	24.134955
NCP/05	Koppie C (Klipfontein)	Kudu	Female	Adult	22 Apr. 2008	3 X *Hippobosca rufipes* flies.	−28.55653	24.01185
NCP/19	Knoffelfontein	Zebra	Male	Adult	Apr. 2008	Carcass unopened. Carcass cremated. Soil collected from site.	−28.71732667	23.76965
NCP/20	Knoffelfontein	Zebra	Male	Adult	Apr. 2008	Carcass unopened. Carcass cremated. Soil collected from site.	−28.77206667	23.789155
NCP/21	Knoffelfontein	Wildebeest	Male	Adult	Apr. 2008	Carcass unopened. Carcass cremated. Soil collected from site.	−28.76305167	23.794065
NCP/22	Knoffelfontein	Zebra	Male	Adult	Apr. 2008	Carcass unopened. Carcass cremated. Soil collected from site.	−28.77310833	23.777205
NCP/34	Knoffelfontein	Zebra	Male	Adult	Apr. 2008	Carcass unopened. Carcass cremated. Soil collected from site.	−28.77241833	23.77861

*Source*: Collated from records of Northern Cape and Skukuza State Veterinary Services, Department of Agriculture, Forestry and Fisheries, South Africa (by authors E.H. Dekker and T. Reardon) NR, no records could be recovered.

Control measures included vaccination of livestock; treatment of the carcass sites which involved spraying either 10% chlorine or 10% formalin on the carcasses; burning of the carcasses and covering up each animal with black plastic or tarpaulins to increase bacterial vegetative cell death and limit blowfly and scavenger access to the carcass. Table 1 indicates the farms affected by the outbreak based on Giemsa stained blood smears as well as carcass condition and observable blowfly, louse fly and crow activity around carcasses.

### Collection of soil and bone samples in 2012 from the 2007–2008 anthrax outbreaks carcass sites

**FIGURE 4:**
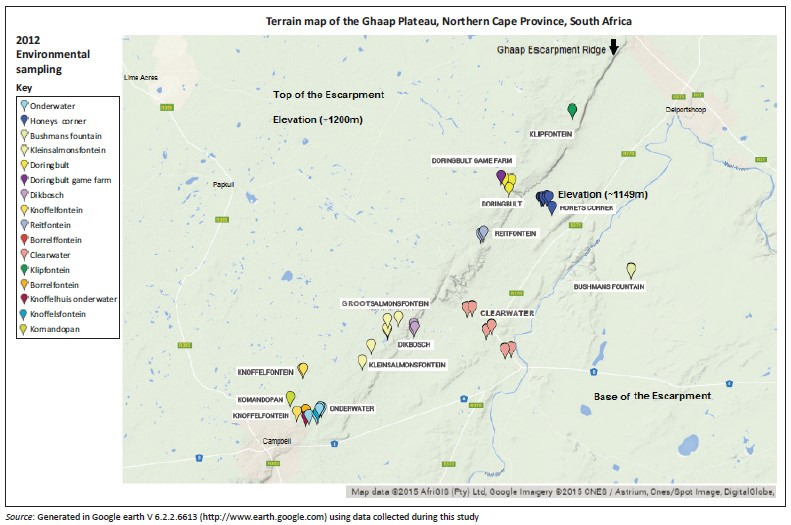
Soil and bone samples collected (*n* = 62) in 2012 from farms and carcass sites identified as anthrax-positive by farmers and State Veterinary Services in 2007–2008, South Africa. Additional soil samples were taken along water runoff paths following the Ghaap escarpment drainage topography (indicated as the diagonal ridge line across the map).

**FIGURE 5:**
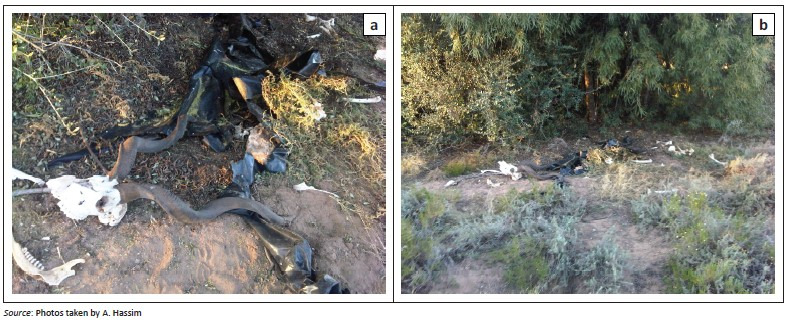
(a) Bones collected in 2012 for isolation of *Bacillus anthracis* from Doringbult game farm, South Africa (Table 1 samples AH56 and AH57). (b) The black plastic tarp covers and remaining kudu bones were clearly visible at this site which was sprayed with 10% chlorine before being covered up with the plastic covers in 2008 (Table 1 sample NC/10).

In March 2012, samples were collected at each of the anthrax positive sites from farms, along the Ghaap Plateau, which suffered losses in 2007–2008 (Table 2). Figure 4 indicates the sites visited. Soil samples were also collected from gravel pits, river and stream beds as well as dry pans where water concentrates after rain. The soil sampling followed the topography (water movement) of the land and thus included areas not sampled in 2008 in the search for concentrator sites (see Figure 1-A1 using the topography of Doringbult game farm as an example). Bone samples were taken at sites where bones were available. About 10 of the 48 sites visited yielded bones from the 2007–2008 outbreaks because the black plastic or tarpaulin remnants or charring from cremation sites were indicators of anthrax cases where control measures were implemented in 2008 (Figure 5). *Bacillus anthracis* positive controls consisted of two bone samples from the original collection in 2008 (NC/14 and NC/29) that were included as bacteriologic isolation controls.

**TABLE 2:** The risk analysis survey of soil and bone sampling of the (2007 and 2008) anthrax-positive carcass sites in 2012.

Location	Processing N°	Soil type	Soil humidity	Soil temperature (°C)	Soil pH	Soil sample depth	N° samples collected	Description	Latitude	Longitude
Doringbult	AH48	Gravel	Dry	23.7	7.58	0 cm – 10 cm	2	Top of drainage basin.	−28.491 415	24.0542567
Doringbult	AH49	Sand	Dry	24.2	7.62	0 cm – 10 cm	2	Site of previous positive carcass.	−28.5007	24.0501467
Doringbult game farm	AH50	Sand	Dry	23.0	7.35	0 cm – 10 cm	2	Site of previous positive carcass.	−28.4901	24.04442
Doringbult	AH51	Humus	Dry	24.3	NA	0 cm – 10 cm	2	Kudu bones previous positively identified carcass. Black tarp cover still on site.	−28.49	24.0444383
Doringbult	AH52	Sand	Dry	24.6	7.61	0 cm – 10 cm	2	Soil under black plastic sail or cover.	−28.49	24.0444233
Doringbult	AH53	Humus/loam	Dry	23.5	NA	0 cm – 10 cm	1	Bone under black tarp sail or cover.	−28.49	24.0444233
Doringbult game farm	AH55	Humus/sand	Dry	24.8	NA	0 cm – 10 cm	2	Bone from second carcass site.	−28.4868	24.0396683
Doringbult game farm	AH54	Sand	Dry	23.7	7.81	0 cm – 10 cm	2	Soil from second carcass site black sail within sight from 2008.	−28.4868	24.0396683
Doringbult game farm	AH56	Humus/sand	Dry	24.1	7.65	0 cm – 10 cm	2	Soil from under the sail of old carcass site.	−28.4868	24.0396683
Doringbult game farm	AH57	Humus/sand	Dry	24.4	NA	0 cm – 10 cm	1	Kudu bone from site under the sail.	−28.4868	24.0396683
Grootsalmonsfontein	AH13	Clay	Dry	23.5	6.96	0 cm – 10 cm	3	Soil collected.	−28.7674	23.7738533
Grootsalmonsfontein	AH16	Sand	Dry	24.0	7.23	0 cm – 10 cm	2	Burning site \ old cremation site.	−28.766	23.7738533
Grootsalmonsfontein	AH14	Clay	Dry	23.8	7.21	0 cm – 10 cm	3	Soil & bone (long bone) of special interest.	−28.7667	23.7742733
Kleinsalmonsfontein	AH1	Humus/loam	Dry	24.2	7.63	0 cm – 10 cm	3	-	−28.7075	23.85113
Kleinsalmonsfontein	AH2	Humus/sand	Dry	23.6	7.12	0 cm – 10 cm	1	-	−28.6886	23.8633483
Kleinsalmonsfontein	AH3	Clay	Dry	23.6	7.81	0 cm – 10 cm	5	Kudu bone + dry clay + soil.	−28.6712	23.8850617
Kleinsalmonsfontein	AH4	Silt	Dry	24.5	7.20	0 cm – 10 cm	1	Dry stream bed.	−28.6691	23.885045
Kleinsalmonsfontein	AH5	Sand	Damp	24.2	7.79	0 cm – 10 cm	1	Gravel pit.	−28.6582	23.8854167
Kleinsalmonsfontein	AH6	Humus/sand	Dry	24.9	7.65	0 cm – 10 cm	2	Carcass sprayed with formalin.	−28.6558	23.9001583
Dikbosch	AH9	Sand	Dry	25.2	6.24	0 cm – 10 cm	1	Soil 1 kg bag for full chemical soil analysis.	−28.6693	23.9209117
Dikbosch	AH8	Gravel	Dry	24.8	6.89	0 cm – 10 cm	2	Stream beds (dry).	−28.6675	23.9222383
Dikbosch	AH7	Clay	Dry	24.1	5.80	0 cm – 10 cm	1	Soil collected.	−28.6639	23.9210117
Klipfontein	AH35	Humus	Dry	24.7	6.89	0 cm – 10 cm	4	35-1 taken from 1 site and 35-2 from few metres away. The 2nd sites are where positive carcass were found in 2008.	−28.4087	24.1350883
Klipfontein	AH35	Humus	Dry	23.1	NA	0 cm – 10 cm	1	Bones.	−28.4087	24.134955
Klipfontein	AH36	Humus	Dry	23.1	NA	0 cm – 10 cm	1	Bones.	−28.5565	24.01185
Knoffelfontein/Onderwater	AH27	Sand	Dry	24.3	5.96	0 cm – 10 cm	2	27-1 Surface; 27-2 20 cm deep. Cremation site	−28.7173	23.76965
Knoffelfontein/Onderwater	AH22	Humus/loam	Dry	24.6	5.43	0 cm – 10 cm	1	Previous site zebra carcass.	−28.7721	23.789155
Knoffelfontein/Onderwater	AH25	Humus	Dry	23.6	NA	0 cm – 10 cm	2	Wildebeest bone.	−28.7631	23.794065
Knoffelhuis Onderwater	AH18	Humus/sand	Dry	23.0	6.08	0 cm – 10 cm	2	Soil & bone (drainage site).	−28.7731	23.777205
Knoffelfontein/Onderwater	AH20	Sand	Dry	23.5	5.28	0 cm – 10 cm	2	Quarry_water drainage.	−28.7724	23.77861
Knoffelfontein/Onderwater	AH21	Sand	Dry	24.3	5.91	0 cm – 10 cm	2	Water drainage.	−28.7721	23.789155
Knoffelfontein/Onderwater	AH23	Sand	Dry	23.5	5.21	0 cm – 10 cm	1	Random low point.	−28.7701	23.7909233
Knoffelfontein/Onderwater	AH24	Sand	Dry	23.7	5.47	0 cm – 10 cm	2	Largest natural water collection site; wide flat pan where water evaporates slowly towards centre.	−28.7637	23.7961233
Knoffelfontein/Onderwater	AH25-1	Sand	Dry	23.5	5.26	0 cm – 10 cm	1	Soil.	−28.7631	23.794065
Knoffelfontein/Onderwater	AH25-2	Sand	Dry	23.4	5.42	10 cm – 20 cm	1	Soil.	−28.7631	23.794065
Knoffelfontein/Onderwater	AH26	Sand	Dry	23.8	5.68	0 cm – 10 cm	3	26-1 Surface; 26-2 10 cm deeper; 26-3 20 cm depth.	−28.7646	23.7928483
Knoffelfontein/Onderwater	AH28	Sand	Dry	24.3	5.64	0 cm – 10 cm	2	Downhill from cremation site.	−28.717	23.7712
Reitfontein	AH36	Clay	Dry	24.0	6.56	0 cm – 10 cm	2	River bed \ drainage gully.	−28.5565	24.01185
Reitfontein	AH37	Gravel	Dry	23.3	6.67	0 cm – 10 cm	2	Second drainage gully.	−28.5557	24.0146167
Reitfontein	AH38	Clay	Dry	24.1	6.82	0 cm – 10 cm	2	Third drainage gully.	−28.554	24.0160717
Borrelfontein	AH15	Humus	Damp	23.2	NA	0 cm – 10 cm	1	Fresh bone.	−28.7667	23.7742733
Borrelfontein	AH17	Sand	Dry	23.8	7.58	0 cm – 10 cm	2	20 m ‘Downstream’.	−28.7661	23.7739983
Mosesberg honeys corner	AH39	Gravel	Dry	23.5	6.89	0 cm – 10 cm	2	Gravel depression.	−28.5125	24.0930583
Honeys corner	AH40	Sand	Dry	23.8	6.96	0 cm – 10 cm	2	Sediment along rocky river bed.	−28.5128	24.0938283
Honeys corner	AH42	Gravel	Dry	24.6	6.84	0 cm – 10 cm	2	Lowest point along fence in river bed.	−28.5131	24.0955783
Honeys corner	AH43	Gravel	Dry	23.1	6.59	0 cm – 10 cm	2	Canal.	−28.5122	24.09575
Honeys corner	AH44	Gravel	Dry	24.4	6.20	0 cm – 10 cm	2	Drainage mound lowest points.	−28.5102	24.1005233
Honeys corner	AH45	Sand	Dry	23.2	7.13	0 cm – 10 cm	2	Stream bed less rocks more sediment as ground levels off.	−28.5124	24.1002383
Honeys corner	AH46	Gravel	Dry	23.6	6.87	0 cm – 10 cm	2	Flood plain.	−28.5116	24.1029517
Honeys corner	AH47	Sand	Dry	23.4	6.28	0 cm – 10 cm	2	Stream bed termination.	−28.5239	24.1073283
Bushmans fountain	AH29	Sand	Dry	24.0	6.46	0 cm – 10 cm	2	Closest to spring source.	−28.5982	24.214687
Bushmans fountain	AH30	Humus	Damp	23.9	6.39	0 cm – 10 cm	1	Outside stream bed.	−28.5985	24.214657
Bushmans fountain	AH31	Sand	Dry	23.7	6.27	0 cm – 10 cm	1	Downstream along stream edge.	−28.5988	24.21477
Bushmans fountain	AH32	Sand	Damp	24.1	6.56	0 cm – 10 cm	1	Fresh wildebeest bone.	−28.599	24.2149
Bushmans fountain	AH33	Sand	Damp	24.0	6.54	0 cm – 10 cm	1	-	−28.5991	24.21489

NA, not applicable.

### Bacterial culture

One gram samples of all soil, crow faeces and ground bone samples were placed in a McCartney bottle with 9 mL sterile phosphate buffered saline (PBS) and shaken for at least 2 hrs. Samples were heat treated at 65 °C for 25 min to select for spores. A 100 µL of this solution was plated out onto Polymyxin EDTA thallous acetate (PET) agar (PLET agar from Turnbull 2008) where lysozyme was omitted (E.H. Dekker, pers. comm., February 2013) and incubated overnight at 37 °C, then further incubated for 24 hrs when colonies were not apparent. Serial 10-fold dilutions for spore counts were made and plated in triplicate for dilutions of 1×10^-1^ to 1×10^-6^. The average colony forming units (CFU) of *B. anthracis* from the 3 plates was calculated. Isolates were confirmed as *B. anthracis* when sensitive to penicillin and γ-phage.

The louse flies’ heads were removed and directly plated onto 5% impala blood agar followed by overnight incubation at 37 °C. All white, non-haemolytic and ‘ground glass’ domed colonies were tested with penicillin and γ-phage to confirm *B. anthracis*. Isolation methods employed in 2012 were exactly the same as those for 2008 with the exception that trimethoprim sulphamethoxazole polymyxin blood agar (TSBPA) (Turnbull 2008) was used in addition to PET and 5% impala blood agar to maximise isolation success.

### Statistical analyses

A cursory examination of the spore data appeared to highlight a disparity in isolate counts from the various bone samples collected. The spore count data from each bone sample were compared to determine if any trends were apparent. Analysis of variance (ANOVA) was used to evaluate carcass spore counts followed by Tukey’s for pairwise comparison test to compare the significance of spore yields between bone samples. Fisher’s exact test was used to determine the significance of carcass effect variables such as locality, species, sex and age because of the small sample size.

## Results

### Isolation and spore counts from 2007–2008 outbreaks

**FIGURE 6:**
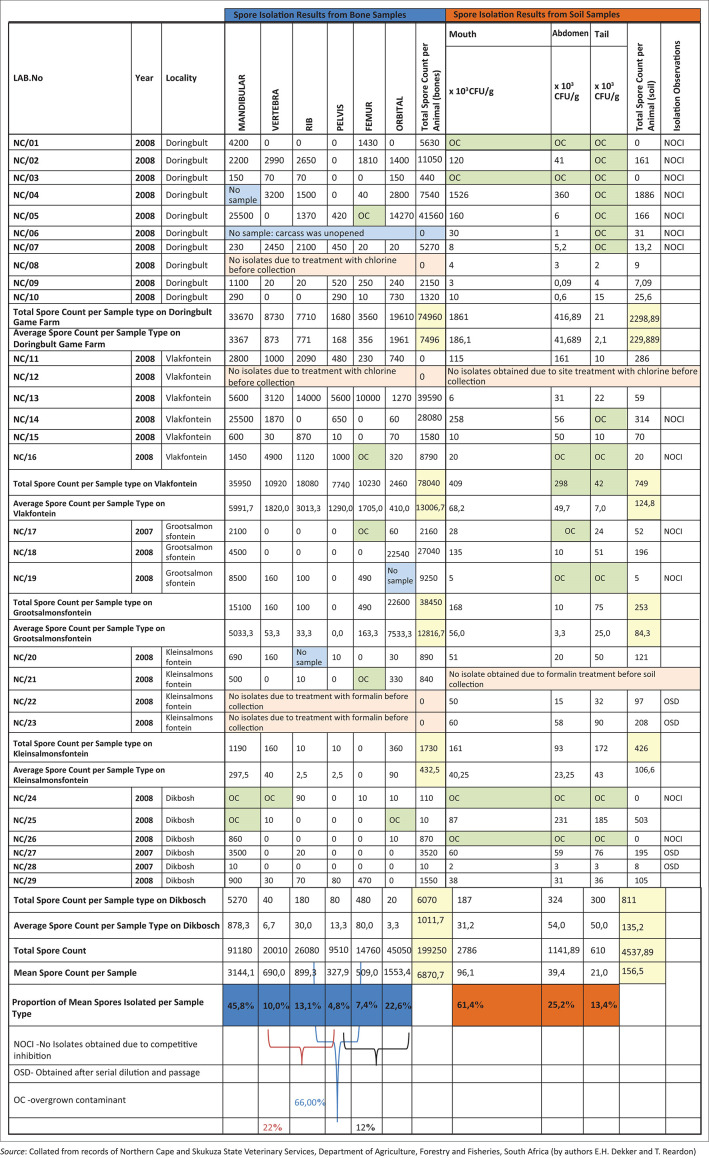
Isolation spore counts for bone samples collected from various accessible parts of a carcass, as well as soil sampled from beneath the mouth, abdomen and head of carcasses from farms along the Northern Cape province, Ghaap Plateau, during the anthrax outbreaks of 2007–2008. The mean spore counts isolated from each bone type, soil location and farm area are indicated in colony forming units per gram.

The first reported cases, where bone samples were collected for bacteriologic diagnostics, were on Dikbosch farm (NC/27 and NC/28) with the isolation of *B. anthracis*. Thereafter, bone and soil samples were collected on six other farms in early 2008 (Table 1) where a mean of 6.87×10^3^ cfu/g and 1.57×10^3^ cfu/g spores from bone and soil samples, respectively were collected at all 29 carcass sites (Figure 6). A mean of 200 spores was isolated from the heads of the louse flies and 300 spores/g from the crow faeces (Skukuza State Veterinary Services laboratory report). The distribution of carcasses was greater on the top of the escarpment (in terms of carcass dispersal) while being confined to the floodplains at the base of the escarpment (Figure 4). Although the first kudus discovered in 2007 were all female, by the end of the anthrax outbreak, overall twice as many male carcasses were discovered than female carcasses. The numbers of confirmed carcasses were 39 kudus, 6 zebra, 2 impala and 2 wildebeest that died of anthrax. During the collection of samples during the 2007–2008 outbreaks, various control measures were taken to decrease the inoculum of spores in the environment as indicated in Table 1. These control methods included spraying carcasses with disinfectants, incineration and covering the carcasses in plastic.

Average *B. anthracis* colony counts from PET and blood agar dilution series plates for the various bone samples can be seen in Figure 6. The ANOVA evaluation of the spore counts had a significant *p*-value of 0.028 between bone types, whereas the Tukey’s pairwise comparison test only showed significance when comparing the femur (*p* = 0.029) and pelvic bones (*p* = 0.049) with the mandibular bone. The mandibular bone generally produced higher spore counts (Figure 6), although, no significance was evidenced by comparison of the mandibular to the rib, vertebrae and orbital bones.

According to Fisher’s scoring, the locality of the carcasses was also significant with a *p*-value of 0.022. The mandibular bone yielded the highest mean spore counts at all localities, with higher counts observed in skeletons or carcasses at Kleinsalmonsfontein followed by Grootsalmonsfontein, Vlakfontein, Doringbult and Dikbosch, respectively (Figure 6; Figure 2-A1). All of these farms are situated on the top of the Ghaap escarpment.

Isolation from the soil under the carcasses at the mouth, abdomen and tail region produced varied spore counts (Table 3). This is reflected in a *p*-value of 0.504 indicating that there is no significance between the sampling location under the abdomen and tail of the carcass (Figure 3-A1). *Bacillus anthracis* spore counts from the soil under the mouth generally yielded higher spore counts accounting for 61% of the mean spores isolated from all the farms (Figure 6). There is an agreement of the *B. anthracis* spore counts between the bone and spore counts in the soil at the location of these bones (mouth, abdomen and tail) (Table 3). The mean spore counts under the mouth or head represented 61% of the total isolated from soil, while the mean spore counts in the mandibular (45%) and orbital (21%) bones together accounted for 66% of the total isolated. The rib (13%) with the vertebrae (9%) made up 22% of the mean spores isolated from bone versus the soil under the abdomen which represented 21% of the total soil spores isolated. The femur together with the pelvic bones comprise 12% of total spores from bone versus the soil collected from under the hind of the animal reflecting 19% of the mean (Figure 6).

**TABLE 3:** *Bacillus anthracis* spore counts obtained from different isolation media from 1 gram of ground mandibular bones collected during the 2007–2008 anthrax outbreaks in the Northern Cape province, South Africa.

Bone sample	*PET agar (2008)	*PET agar (2012)	**TSPBA (2012)	Blood agar (2012)
NC/14 Mandible	25 500	18 000	17 000	26 000
NC/29Mandible	900	550	500	750

* , Polymyxin EDTA thallous acetate;

** , Trimethoprim sulphamethoxazole polymyxin blood agar.

Random soil samples taken from two pans at Doringbult did not yield any *B. anthracis* spores, whereas random samples collected at the lowest points downhill on Doringbult of carcasses NC/01 and NC/04 yielded 5×10^4^ and 1.2×10^4^ spores per gram of soil on PET agar, respectively (Figure 1-A1). A soil sample collected at Grootsalmonsfontein at the lowest point below carcass NC/18 contained 400 spores per gram of soil.

### Isolations in 2012

The soil and bone samples collected in 2012, yielded a variety of *Bacillus* spp. on the different media employed including *Bacillus subtilis, B. cereus* and *Bacillus thuringiensis,* but contrary to expectation, no *B. anthracis* was found. The control *B. anthracis* bone samples NC/14 and NC/29 from the original 2008 outbreaks were positive for the presence of *B. anthracis* spores as indicated in Table 3. Similar *B. anthracis* spore counts were obtained in 2008 and 2012 from the reference bone samples NC/14 and NC/29.

## Discussion

The isolation of *B. anthracis* from samples collected in 2007–2008 demonstrates a unique ecology at each carcass site with a variety of vectors such as water, crows, blowflies and louse flies evidencing activity. There is also a difference in *B. anthracis* yields between bone types and soil samples collected from the carcass sites during this outbreak. The mandibular bone yielded the highest spore counts and mean spore counts under the mouth or head of the carcass yielded the highest counts from soil. Control measures were taken during the 2008 anthrax outbreak seemed to reduce the spore counts as no *B. anthracis* could be isolated from the soil or bone from the carcass sites in 2012.

During the anthrax outbreaks of 2007–2008 in NCP, a disproportionate number of adult male kudus died from the disease. This is in keeping with studies in Etosha (Lindeque & Turnbull 1994). This is notable because kudus are browsers and not dependent on as frequent water intake as their grazing counterparts. Kudu seemed to be the most susceptible species which died of anthrax, followed by zebra, impala and wildebeest, all of which except the kudu died on the game conservancies. The carcass distribution is not well represented because State Veterinary Services only intervened on the farms and not the national parks and nature conservancies where the disease is considered part of the natural ecology. On the other game farms, domestic animals and kudu died in greater numbers during the 2007–2008 anthrax outbreaks in NCP.

It has been consistently noted that spore counts in the soil appear to be dependent on the opening of a carcass and soil closest to these openings produce higher contagion concentrations (Dragon et al. 2005). This can be clearly seen for soil spore counts taken from under carcasses in Doringbult with the highest spore counts obtained from soil under the mouth of 2 female kudu carcasses (Figure 5 samples N/C04 and NC/05). Both of these carcasses had opened heads. The 2 carcasses did differ in spore counts obtained from soil under the abdomen where NC/04 had a noticeable hole in the abdomen while no such observation was made for NC/05. According to Bellan et al. (2013), the spore density at a carcass site is dependent on the vegetative cell concentration at host death coupled with the sporulation efficiency of the bacterium, spore survival and propensity for environmental replication. Sporulation is triggered by a paucity of nutrients coupled with exposure to oxygen (Koehler 2009; Minett & Dhanda 1941; Sterne 1937; Turnbull 2008; Van Schaik, Prigent & Fouet 2007). As such, soils contaminated by bacterial laden bodily fluids would contain more spores than an area a distance away from the carcass, which was observed by Dragon et al. (2005). The higher spore counts obtained from soil under the mouth of carcasses because of haemorrhagic discharge affirms the findings of Bellan et al. (2013), Dragon et al. (2005) and our study where high spore count were obtained from carcass heads opened by scavengers, thus providing extravasation fluid as enrichment media to the soil for possible vegetative bacterial amplification in the environment.

Statistically the mandible, ribs, orbital bone and vertebrae produced equivalent *B. anthracis* spore counts, although the mean spore counts demonstrated better isolation success rates from the mandibular, rib and orbital bones. In the NCP, crows are often observed to peck out the eyes of a carcass (Hugh-Jones & De Vos 2002). Because of the lack of blood clotting (Leppla 1984) and the exposure of the haemorrhagic fluid in the orbital sockets to the elements; sporulation would be triggered along with increased contagion deposition in the environment. In KNP scavengers like vultures and hyeana are responsible for opening the carcass (Hugh-Jones & De Vos 2002). Because of a lack or limiting numbers of scavengers in NCP, crows as well as scavengers like jackals and to a lesser extent hyena and leopards are responsible for opening the carcass.

Blowflies as well as louse flies have been observed on carcasses in NCP (Hugh-Jones & De Vos 2002). Louse flies belong to the family *Hippoboscidae* and are blood-feeding flies. These flies although strong fliers, seldom fly more than a few metres, choosing instead to move to the next closest host when disturbed. They are abundant in the summer months and can be found clustered on the perineal and pubic regions as well as the neck and sides of the animal. They have a long standing association with anthrax transmission amongst cattle (Howell, Walker & Nevill 1978). Cattle louse flies were observed on kudu carcasses at sites in Clearwater (Schmidtsdrif) and Dikbosch in NCP during the 2007–2008 outbreaks. The louse flies were easily collected from the kudu carcasses and an average of 200 spores were isolated from the head (mouthparts) of a louse fly. As a blood feeder, it has been suggested that this makes it a potential mechanical vector to its next animal host (De Vos & Turnbull 2004; Howell et al. 1978). This however requires further study.

The ability of anthrax spores to persist in the earth for extended periods is because of specific soil and climatic conditions, which provide an environment conducive to its survival (De Vos & Turnbull 2004; Smith et al. 2000). Soils rich in organic matter, ample in calcium, prone to alkalinity and with an ambient temperature above 15.5 °C are considered optimal for persistence of spores. The exosporium of the spore is negatively charged (this charge and its strength are pH dependent) whereas humus particles are positively charged and are therefore able to chelate, which then provides environmental stability for the spores (Hugh-Jones & Blackburn 2009). The spores can persist in soil and bone for decades or even centuries under such conditions (De Vos 1998; Wilson & Russell 1964). These humus-spore clumps have the added advantage of being buoyant. During flooding, as was the case of the NCP 2007–2008 anthrax outbreaks, these clumps can be deposited or concentrated at water collection sites once the water evaporates, as the spores are highly resistant to UV radiation (Hugh-Jones & Blackburn 2009; Hugh-Jones & De Vos 2002; Vilas-Boas et al. 2007).

Many studies have evaluated the influencing factors on the dissemination and survival of *B. anthracis* that leads to outbreak trends. These include stocking rates, elevation, soil factors as mentioned above, water content and rainfall (Barro et al. 2016; Blackburn 2010; Chikerema et al. 2013; Joyner et al. 2010). Steenkamp (2013) elucidated the importance of the role of water and topography in the dissemination of anthrax in KNP along with various other studies (Pienaar 1960; Viljoen et al. 1928). The Ghaap is unique in its distribution of natural springs, Kimberlite pipes and dolomitic sinkholes which serve to flush the area with groundwater during periods of heavy rainfall and after groundwater recharge (Smit 1978). This theory is consistent with the high spore counts observed in water collection pans downhill from carcass sites during the 2007–2008 anthrax outbreaks.

It can be argued that *B. anthracis* survival in the soil and its subsequent exposure to inclement conditions could dilute the spores to a negligible detection limit (Smith et al. 2000), however, according to De Vos (1998) spores can remain stable in bone for decades if not centuries. The stable spore counts from the stored bone samples isolated in 2012 (Table 3) are an example of the spores endurance. The PET agar plates used in this study had spore counts equivalent to the TSPBA. The lack of *B. anthracis* spores from bone samples collected in 2012 reinforces the effectiveness of control measures employed during the 2007–2008 outbreaks. Because of the employment of a variety of control measures, the efficacy of the individual actions of using chlorine, formalin or the black sails or tarps cannot be determined, nor whether all the measures worked synergistically to reduce the inoculum in the environment. It is unclear to what extent the control measures of the 2007–2008 outbreaks managed to further stem the dissemination of spores through dilution in the environment. Bones treated at carcass sites were not a source of infection to animals suffering from pica in later seasons because they did not yield viable spores which could pose a threat of infection (De Vos 1998; De Vos & Turnbull 2004).

The reduction of inoculum in the environment and the corresponding reduced spore exposure is paramount in the control of anthrax (De Vos & Turnbull 2004; Turnbull 2008; Watson & Keir 1994). This was indicated when 2012 isolations from the reference bone samples collected during the outbreak (used as positive controls) had spore counts similar to those enumerated at the time of collection.

## Conclusion

There are too many variables at each carcass site that could possibly influence the dissemination of anthrax which serves to complicate our understanding of the disease as seen with the 2007–2008 anthrax outbreaks in NCP. Modelling which includes all the factors (host, bacterium, vectors and environment) will provide more insight into the unique ecology of anthrax on the Ghaap Plateau.

Carcass bones are a reliable source for the successful isolation of *B. anthracis*. The mandibular and orbital bones as well as soil beneath the head of a carcass proved to be a prodigious source of viable *B. anthracis* spores from carcasses collected during the 2007–2008 NCP outbreaks

The control measures of burning or spraying carcasses with chlorine or formalin and then covering them with tarp or plastic were applied by farmers on the recommendation of the veterinary services during the 2007–2008 outbreaks. As no spores could be isolated in 2012 from the same sites and bones remaining in the environment, this finding indicates that these control measures had greatly reduced or possibly even eliminated the inoculum at the treated carcass sites.

## Figures and Tables

**FIGURE 1-A1:**
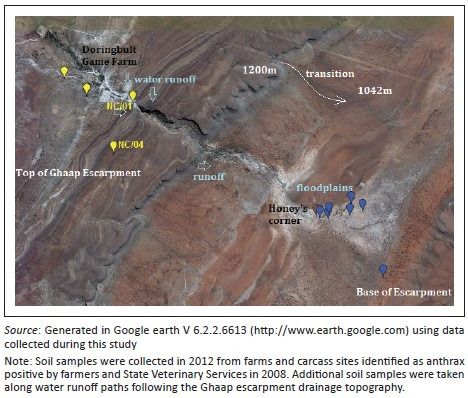
A closer view of water drainage on Doringbult and Honey’s Corner farms in the Ghaap Plateau in the Northern Cape province. The water runoff moves towards Doringbult and thereafter to Honey’s corner farm where mass alluvial deposition occurs (white areas).

**FIGURE 2-A1:**
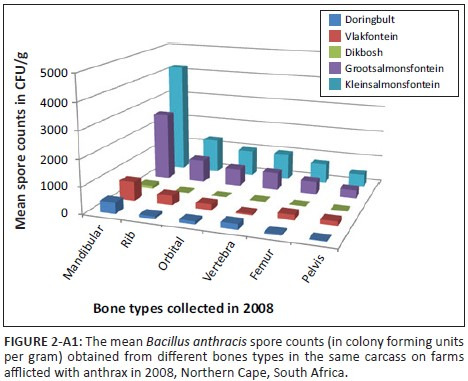
The mean *Bacillus anthracis* spore counts (in colony forming units per gram) obtained from different bones types in the same carcass on farms afflicted with anthrax in 2008, Northern Cape, South Africa.

**FIGURE 3-A1:**
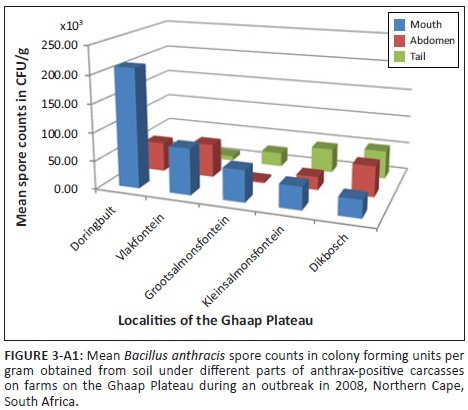
Mean *Bacillus anthracis* spore counts in colony forming units per gram obtained from soil under different parts of anthrax-positive carcasses on farms on the Ghaap Plateau during an outbreak in 2008, Northern Cape, South Africa.
